# Supplementary material for: Induced Aggregation of Epoxy Polysiloxane Grafted Gelatin by Organic Solvent and Green Application
Source: Molecules. 2019 Jun 18;24(12):2264. doi: 10.3390/molecules24122264 (PMC6630429; doi:10.3390/molecules24122264)
Supplement: Supplementary file 1 [file molecules-24-02264-s001.pdf]

Supporting Information

Induced Aggregation of Epoxy Polysiloxane Grafted Gelatin by Organic Solvent and Green Application

Zhen Zhang [1], Dongmei Zhang [2], Huijun Ma [1], Jing Xu [1,*], Tianduo Li [1], Zhaoning Cai [1], Haifeng Chen [1], Jinghui Zhang [1] and Hao Dong [1]

[1] Shandong Provincial Key Laboratory of Molecular Engineering, School of Chemistry and Pharmaceutical Engineering, Qilu University of Technology (Shandong Academy of Sciences), Jinan 250353, PR China; zhangzhen950305@163.com (Z.Z.); 17862182055@163.com (H.M.); litianduo@163.com (T.L.); caizhaoning1221@163.com (Z.C.); chenhaifeng529@163.com (H.C.); zjh804300441@163.com (J.Z.); d237658934@163.com (H.D.)

[2] Shandong Institute for Food and Drug Control, Jinan 250101, PR China; zhangdm1000@163.com

* Correspondence: xujing77611@163.com

CONTENTS

1. Preparation of epoxy polysiloxanes

The synthesis of epoxy siloxane is divided into two steps. The first step is the synthesis of a single Si-H terminated polysiloxane (PDMS-H), with D3 as a monomer, and n-butyllithium as an initiator. Benzene is used as the solvent, tetrahydrofuran as promoter, and dimethyl-hydrogen-silicic chloride as capping agent; using active anionic polymerization technology, narrow distribution PDMS-H was synthesized by controlling the molar ratio between n-BuLi and D3. The synthesis reaction is as follows：

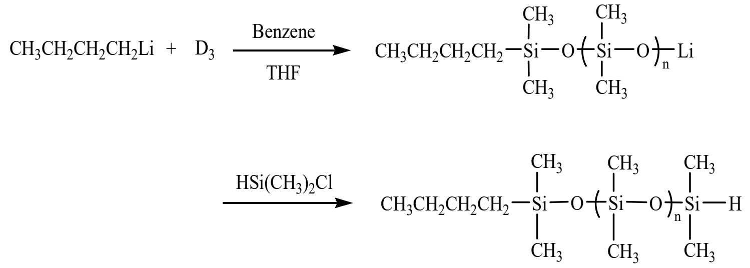

Figure S1. Preparation of PDMS-H

The second step is the hydrolyzation reaction of allyl glycidyl ether (AGE) with PDMS-H under the catalyst chloroplatinic acid conditions to produce epoxysiloxane (PDMS-E)

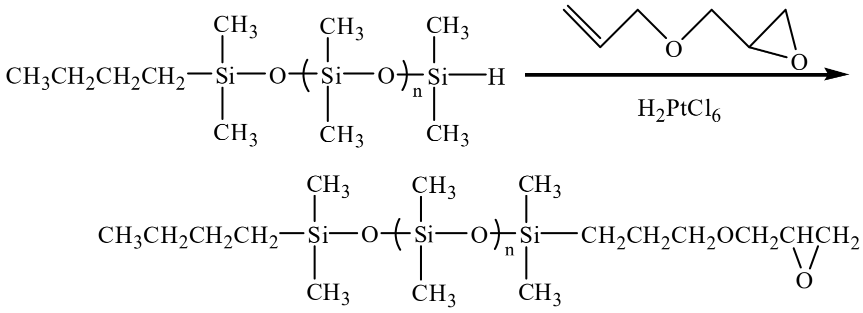

Figure S2. Preparation of PDMS-E

2. IR spectroscopy characterization

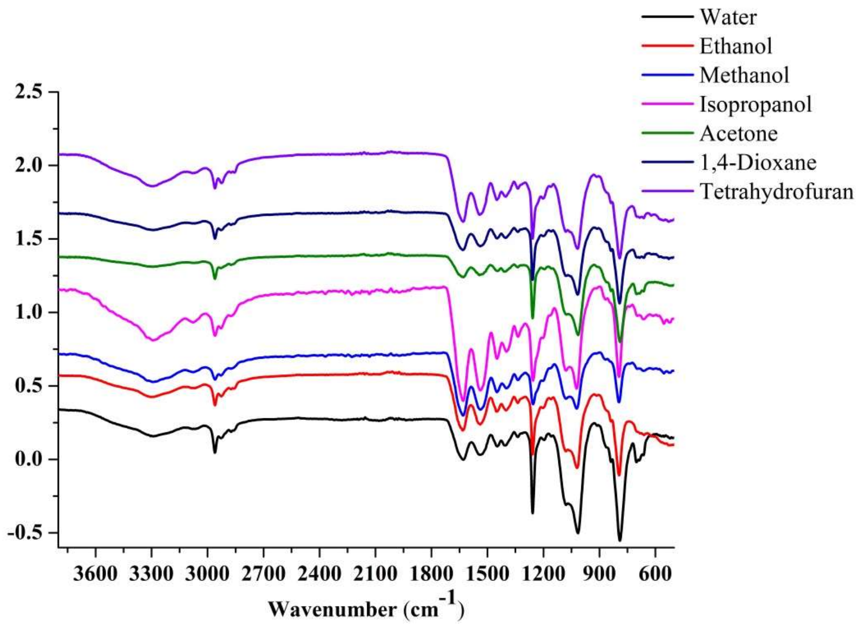

Figure S3. IR spectra of blank and solvent-polymer films.
